# University of Louisville

**Published:** 1851-04

**Authors:** 


### THE WESTERN JOURNAL.

THIRD SERIES.—VOL. VII.—NO. IV.

LOUISVILLE, APRIL 1, 1851.

### UNIVERSITY OF LOUISVILLE.

The annual commencement in the Medical department of the University of Louisville was held on the evening of the first day of March, on which occasion the degree of M. D. was conferred on eighty-one students who had passed satisfactory examinations before the medical faculty. The honorary degree of M. D. was at the same time conferred on Dr. Edward B. Haskins, of Clarksville, Ten. The Valedictory Charge to the graduates was delivered by Prof. Yandell. The whole number of students in this department of the University of Louisville during the last session, was 282.

Our readers at a distance may have had their attention drawn by the discussions in the city newspapers to a proposed change in the mode of choosing the trustees of the University of Louisville, which, it has been feared, might jeopard the prosperity of this institution. The new city charter lately passed by the Legislature provides for the election of trustees by the people, but in as much as many members entertained doubts respecting the constitutionality of this clause in the charter, it was enacted that it should not take effect until the question had been adjudicated by the Jefferson Circuit Court. If the decision of the Court should be adverse to the new charter, the trustees will continue to be appointed as now.

If the charter should be decided to be constitutional, and a board of trustees be elected in a period of excitement, some of the friends of the school are apprehensive of consequences the most injurious to its prosperity. It is impossible to predict what the result would be, but it is proper to state that in the opinion of many of its most judicious friends no mischief is likely to follow such a change. A belief is confidently entertained, that a wise and conservative board would be elected by the people, and that the institution would continue to prosper as it has done under the able administration of the trustees who have had control of its affairs. It is to be remembered that we live in a democracy, and, particularly, that the medical school of Louisville was founded and endowed by its citizens; and if the people elsewhere and in other elections have been found capable of choosing wisely and well, we do not see any reason to doubt that they will in this case, if the duty be imposed upon them, select the men best qualified to guard and advance their interests. Demagogues, it is said, and men seeking places in the school, will mislead the people and cause the board to be filled by radicals and disorganizers, in the hope that in the revolutions that must ensue they may stand a chance to come in. This is certainly the danger to be apprehended, but it is not peculiar to such an election, and as it has been generally averted in States where all offices, from that of supreme judge to that of town constable, are filled by the people, let us hope that the same conservative principles will prevail there when every election shall have become popular.

For ourselves, we are free to declare that, whatever be the mode of electing its trustees, we expect the medical school of Louisville to continue to flourish. It has been planted too securely, and has established itself too firmly in the confidence of the profession to be seriously affected by these transient local agitations. We should be sorry to see its present excellent board of trustees superseded, but if such should be the decision of the courts we shall still hope to see a judicious board elected by the people. It is the interest of a few individuals, if they cannot obtain possession of its places, to destroy the school, and they conceive that there is no better way of accomplishing their ends than to keep up this agitation; but it is the interest of the citizens to protect and preserve an institution which is one of the sources of the city's prosperity, and they will not fail to do it. This contest has been going on, unintermittedly, for eleven years, and yet all this time the school has grown and flourished in a manner remarkable even for our country of rapid development. Under one form and another, but still by the same selfish, disappointed persons, it has been continued from year to year, but without any effect upon the prosperity of the school. We suppose the war will still be renewed however the present question may be decided, and the school, no doubt, will go on to prosper as heretofore.

